# The Clinicopathologic Spectrum of IgG4-Related Disease

**DOI:** 10.4274/balkanmedj.2018.0809

**Published:** 2018-07-24

**Authors:** Ifeyinwa Emmanuela Obiorah, Alicia Henao Velasquez, Metin Özdemirli

**Affiliations:** 1 Department of Pathology, Medstar Georgetown University Hospital, Washington, USA

**Keywords:** Autoimmune, diagnosis, IgG4, IgG4-related disease, pathology, treatment

## Abstract

Immunoglobulin G4-related disease is a fibroinflammatory systemic disease that is characterized by focal or diffuse organ infiltration by immunoglobulin G4-bearing plasma cells. Immunoglobulin G4-related disease may affect any organ, and a high index of suspicion is necessary for early detection to avoid irreversible fibrosis, organ dysfunction, and death. Tumor-forming lesions are common radiological features of immunoglobulin G4-related disease that need to be differentiated from malignancies. The diagnostic approach requires the integration of clinical, biochemical, and radiographic manifestations with classic histopathologic features, which remain crucial to diagnosis. The histology of immunoglobulin G4-related disease is determined by a dense lymphoplasmacytic infiltrate, storiform fibrosis, and obliterative phlebitis in the presence of increased immunoglobulin G4-positve plasma cells. Although immunoglobulin G4-related disease forms a distinct, clinically independent disease category, many questions and problems remain unanswered, especially on its pathogenesis and the role of immunoglobulin G4. Advances in the understanding of immunoglobulin G4-related disease are likely to change the diagnostic approach in the future and create potential targets for therapeutic purposes. Here we describe the concept of immunoglobulin G4-related disease and the most recent knowledge in the clinico-pathological characteristics on this emerging disease. This study can guide clinicians in early diagnosis and prevent unnecessary surgical resections.

Immunoglobulin G4 (IgG4)-related disease (IgG4-RD) is a chronic immune-mediated disease that can affect any organ in the body. IgG4-RD was first described by Sarles et al. (1) in 1961 in a case of pancreatitis with hypergammaglobulinemia. In 1967, Comings and coworkers (2) reported a case of familial multifocal fibrosclerosis, which presented with features of retroperitoneal fibrosis, mediastinal fibrosis, sclerosing cholangitis, Riedel’s thyroiditis, and pseudotumor of the orbit; these symptoms were all thought to be different manifestations of one disorder. In 2003, the extrapancreatic manifestations of IgG4-RD were first described by Kamisawa et al. (3) among a population of patients with autoimmune pancreatitis; this result suggested that multifocal fibrosclerosis in these patients were part of the same clinicopathologic spectrum. IgG4-RD tends to affect middle-aged to elderly individuals with a predilection for the male sex (4,5). However, studies demonstrate a wide age range that includes pediatric patients (6). The pathogenesis of IgG4-RD remains largely unknown, and the exact role of the IgG4 molecule has not been identified. Data support that the inflammatory and fibrotic processes that drive IgG4-RD are initiated by a combination of T-helper type 2 (Th2) cells and regulatory T cells (Treg cells) (7). The disease entity can affect virtually any organ including the pancreas, liver, biliary system, salivary glands, lacrimal glands, retroperitoneum, lymph nodes, and kidney (6,8), typically presenting as a mass or enlargement of one or more organ, which may mimic a malignancy (8). The diagnosis of IgG4-RD is vital to prevent unwarranted surgical resections, and it usually involves a combination of clinical, radiological, serological, and histopathological findings. Although most patients with IgG4-RD have an elevated serum IgG4 concentration, at least one-third of patients have a normal serum IgG4 concentration. Therefore, an elevated serum IgG4 is neither sensitive nor specific for the diagnosis and has a general role as a screening tool (5). The mainstay of diagnosis, regardless of the organ involved, is the identification of histopathologic features, which include dense lymphoplasmacytic inflammation, storiform fibrosis, obliterative phlebitis, and demonstration of dominance of IgG4-positive plasma cells (9). IgG4-RD can be challenging to diagnose when a less frequently affected organ is involved, and a high index of clinical suspicion is needed. This paper describes the potential pathogenesis and clinical, radiologic, and histopathological features of IgG4-RD and review responses related to therapy.

## PATHOPHYSIOLOGY OF IMMUNOGLOBULIN G4-RELATED DISEASE

IgG4 is the least abundant in the IgG subclass and accounts for <5% of the total IgG in healthy individuals (10). The overabundance of IgG4 antibodies may be due to the antibodies behaving as tissue-destructive immunoglobulins or excess IgG4 may simply be a response to an unknown primary inflammatory stimulus (6). IgG4 molecule is not believed to be a driver of pathogenesis because it is involved in a continuous process of half molecular exchange, which is also known as Fab-arm exchange. This process involves the dissociation of the heavy chain dimers of an IgG4 molecule, and each half-molecule is associated with another different hemi-IgG4 protein (5,10). Most secreted IgG4 molecules behave as monovalent, non-cross-linking antibodies and cannot form large immune complexes or directly fix complements. Thus, IgG4 molecules are generally thought to be non-inflammatory, and they act to dampen rather than to incite or accelerate chronic immune activation. This function correlates with the well-known association of IgG4 with immunoglobulin E (IgE)-mediated allergy, where IgG4 antibodies induced by allergy treatments induce tolerance and protect allergic subjects from anaphylactic reactions by competing with allergen-specific IgE (11).

The exact mechanism of IgG4-RD remains unknown. One proposed mechanism is linked to Th2 cells, which are involved in the regulation of IgG4 production. Th2 cells secrete interleukins (IL-4, IL-5, and IL-13), which activate B cells to switch to IgG4 and IgE (5). Mattoo et al. (12) demonstrated that circulating Th2 memory cells accumulate among patients with IgG4-RD who have preexisting histories of atopy but not among those without atopic disease. This finding is unsurprising given that eosinophilia and increased serum IgE, which occur in 40% of patients with IgG4-RD, are mediated by Th2 cytokines (13). However, the precise mechanism of Th2-mediated IgG4-RD needs further clarification. Tregs may play a role in IgG4-RD, which is suggested by showing that Foxp3 messenger RNA, a transcription factor specific for CD4+ CD25+ Tregs, is significantly increased in IgG4-related pancreato-cholangitis in comparison with primary sclerosing cholangitis and primary biliary cirrhosis (14). In addition, Treg- mediated expression of IL-10 and transforming growth factor-β are overexpressed in IgG4-RD. IL-10 is implicated in the B cell-induced production of IgG4 (15), and transforming growth factor-β is a fibrogenic cytokine that may be involved in the promotion of fibrosis in IgG4-RD. Another potential mechanism is the induction of CD4-positive T cells, which stimulate immune cells such as macrophages, myofibroblasts, and fibroblasts to drive fibrosis; this condition is presumably sustained by antigen-presenting B cells (5). This result may lead to the substantial improvement in IgG4-RD following treatment with anti-CD20 therapy. Further evaluation of the immune-mediated pathway of IgG4-RD is necessary to generate potential targets that can result in the elimination of IgG4-mediated inflammation and fibrosis.

## CLINICAL MANIFESTATIONS

The clinical presentations of IgG4-RD are widely variable and can manifest in one or more organs synchronously or metachronously. IgG4-RD usually presents as a subacute or chronic process, and it can range from mild localized symptoms to major tissue damage and subsequent organ failure. Most patients are not constitutionally ill, so fever, malaise, night sweats, or weight loss are unusual (16). Tumorous lesions and allergic disease are common findings observed in IgG4-RD (6). One-third of patients with IgG4-RD have atopic disease, peripheral blood eosinophilia, and elevated IgE levels (17,18). Nevertheless, a subset of nonatopic patients have peripheral blood eosinophilia and elevated IgE, suggesting that these conditions may be related to IgG4-RD itself rather than atopy. The most commonly reported manifestations are those related to the mass effect of pseudotumors in the affected organs or adjacent surrounding structures (19).

### Imaging Features

Imaging is usually the first diagnostic tool to identify the tumorous swellings that occur in patients with IgG4-RD. Computed tomography (CT) may delineate a focal or diffuse pseudotumoral swelling of organs and identify the extent of multiorgan involvement. However, multiorgan involvement may be better assessed by 18F-fluorodeoxyglucose positron emission tomography/CT (18F-FDG PET/CT) (20,21). Imaging with 18F-FDG PET/CT enables delineation of the inflammation sites by identifying hypermetabolic activity to evaluate the extent of the disease, guide biopsy decision, and monitor response to treatment (22). Magnetic resonance imaging (MRI) of affected organs generates a low signal on T2-weighted imaging, with homogeneous enhancement reflecting increased cellularity and fibrosis (23). Imaging in patients with IgG4-RD typically reveals nonspecific findings, which are insufficient to differentiate the disorder from neoplastic conditions that present with mass-forming lesions. The exception to the rule is autoimmune pancreatitis, which shows classic CT or MRI findings of a diffusely enlarged sausage-shaped pancreas with a halo of edematous tissue and is pathognomonic for the disease entity (24). The major organ radiographic findings of IgG4-RD are summarized in Table 1.

## ORGAN MANIFESTATION

### Pancreas

**Figure 1:**
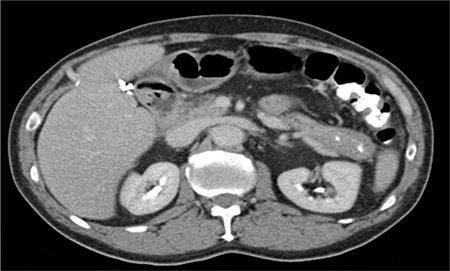
Computed tomography scan of the pancreas. The image demonstrates a hypoattenuating pancreatic mass.

The pancreas was the first organ associated with increased serum levels of IgG4 (3,25). Two subtypes of autoimmune pancreatitis (AIP) have been described. Type 1 AIP is associated with IgG4-RD and characterized by the classic histopathological findings of lymphoplasmacytic sclerosing pancreatitis (26). Type 2 AIP is not related to IgG4-RD and is recognized based on the identification of neutrophilic infiltrate into the pancreatic duct epithelium (27). The most frequent clinical manifestation of AIP is painless obstructive jaundice, which can mimic pancreatic cancer in the presence of pancreatic enlargement or mass lesion (Figure 1) (28). Endoscopic ultrasonography-guided fine needle aspiration can be attempted to exclude pancreatic cancer and should be performed before any empirical trial of steroids is undertaken. Less common clinical presentations include focal pancreatic mass, diffuse pancreatic enlargement, and pancreatic duct stricture (29). A minority of patients can present with severe abdominal pain with increased lipase levels, hence mimicking acute pancreatitis, or may present with features of chronic pancreatitis (6). In addition, some patients present with acute glucose intolerance, and treatment with corticosteroid improves glycemic control in a subset of patients (30). A characteristic feature of AIP, which belongs to the spectrum of IgG4-RD, is the involvement of other organs including bile duct stricture, renal involvement, retroperitoneal fibrosis, orbital pseudotumor, and diffuse lymphadenopathy (28,29). These symptoms can serve as important supportive diagnostic clues. Although radiologic features of AIP are usually characteristic and may not warrant histologic diagnosis, the finding of a normal pancreas on imaging does not preclude AIP and requires further investigation.

### Hepatobiliary System

IgG4-related sclerosing cholangitis is commonly associated with type 1 AIP. Solid mass lesions in hilar and perihilar ducts show close resemblance to primary sclerosing cholangitis and cholangiocarcinoma (31). Given that serum IgG4 concentrations nor cholangiographic or cholangioscopic features do not differentiate these disorders clearly (32,33,34), endoscopic biopsy is often required to differentiate IgG4-related sclerosing cholangitis from both primary sclerosing cholangitis and hilar cholangiocarcinoma. Histopathology is crucial for differential diagnosis. IgG4-related hepatopathy has been described in patients with type 1 AIP and autoimmune hepatitis, or manifest as pseudotumors in the liver parenchyma (35).

### Salivary Glands

Mikulicz’s disease, which consists of symmetrical swelling of the lacrimal and salivary glands, is now recognized as IgG4-related dacryoadenitis and IgG4-related sialoadenitis (IgG4-RS) (6). Bilateral submandibular gland involvement is the most frequent presentation of IgG4-RS. However, the parotid, sublingual, and labial salivary glands may also be affected (36). IgG4-RS was previously considered to be a subset of Sjögren’s syndrome (SS). Despite their similarities in organ involvement, IgG4-RS and SS are quite different conditions and need to be differentiated from each other. A comparison between IgG4-RS and SS reveals the following (8,37): in contrast to IgG4-RS, the enlargement of the parotid gland is predominant in SS. Patients with IgG4-RS have less symptoms of xerophthalmia, xerostomia, or arthralgia than patients with SS. Patients with IgG4-RS have coexisting AIP, interstitial nephritis, allergic rhinitis, and/or bronchial asthma, but anti-SS-A and anti-SS-B antibodies, rheumatoid factor, and anti-nuclear antibody are negative in most patients. Elevated serum IgG4 and marked infiltration with IgG4-positive plasma cells in IgG4-RS marks the most important difference between IgG4-RS and SS. Finally, glucocorticoid therapy is highly effective in patients with IgG4-RD but has limited effect in patients with SS. Given that the radiographic findings of IgG4-RS are nonspecific, differentiation of multiglandular and localized involvement from malignant lymphoma for multiglandular disease and salivary gland carcinoma for localized disease is challenging.

### Orbits

IgG4-RD can cause orbital inflammation that involves the lacrimal glands, extraocular muscles, orbital soft tissues, sclera, nasolacrimal duct, and trigeminal nerve; it is sometimes accompanied with bone destruction and development of a saddle nose deformity (38). Unlike systemic IgG4-RD, patients with IgG4-related orbital disease (IgG4-ROD) affect both male and female sexes equally (39). Patients with IgG4-ROD often present with painless bilateral orbitopathy. The clinical symptoms are indolent and often occur as a result of regional mass effect or diffuse infiltration of involved tissues, including painless proptosis, diplopia, and impaired eye movements (40). IgG4-ROD is usually bilateral than unilateral at presentation. By contrast, patients with “orbital pseudotumor” or “idiopathic orbital inflammation,” which is not related to IgG4, tend to be unilateral and usually present with an acute onset of pain, erythema, proptosis, and restriction of ocular motility (41). Nonetheless, a considerable number of cases may be IgG4-related ophthalmic disease. Other considerations in the differential diagnosis of patients with IgG4-ROD include SS, granulomatosis with polyangiitis, sarcoidosis, lymphoma, infection, Graves’ thyrotoxicosis, and cancer (38).

### Ear, Nose, and Throat

Sinonasal manifestations of IgG4-RD, although rare, have been reported and are now a recognized entity with two patterns of presentation (40). Patients may present as a destructive locally invasive mass, which can manifest as nonspecific symptoms such as nasal obstruction, rhinorrhea, epistaxis, facial pain, and anosmia (42,43,44). Frequent sites of involvement include the maxillary sinus, nasal cavity, and septum, whereas the sphenoid sinus and anterior ethmoid air cells are less commonly affected. Sinonasal IgG4-RD has a predilection for bone destruction, thereby mimicking sinonasal lymphoma (45). The second pattern of sinonasal IgG4-RD is diffuse infiltration of the sinonasal mucosa with an IgG4-positive plasmacytic infiltrate. This manifestation closely resembles “idiopathic chronic rhinosinusitis,” which also demonstrates IgG4 plasma cells in the mucosa, thereby making differentiation between the two disorders very difficult (46). As previously noted, allergic features occur in a substantial subset of patients with IgG4-RD, so an allergic pathophysiology may be responsible for the prevalence of IgG4-related chronic rhinosinusitis in these patients (47). Otologic involvement with IgG4-RD can manifest as otitis media with effusions, eosinophilic otitis media, and sensorineural hearing loss. Takagi et al. (48) reported five cases that responded to glucocorticoid therapy. Although the patients had biopsy-proven IgG4-RD at other sites, none of the otologic cases were confirmed by biopsy.

### Thyroid

Thyroid gland IgG4-RD includes Reidel’s thyroiditis and fibrosing Hashimoto’s thyroiditis. Reidel’s thyroiditis is a chronic fibrosclerotic disorder that was first described by Reidel (49) in 1896; it manifests as a hard infiltrative lesion in the thyroid gland. Dahlgren and coauthors (50) demonstrated increased IgG4-positive plasma cells and the morphologic features of IgG4-RD in patients with Reidel’s thyroiditis. The fibrosing variant of Hashimoto’s thyroiditis is now considered part of the clinical spectrum of IgG4-RD, and it is characterized by dense bands of fibrosis and marked infiltrations of IgG4-positive plasma cells (36). Compared with Riedel’s thyroiditis, extra-thyroidal fibrosis is absent in fibrosing Hashimoto’s thyroiditis. In addition, patients with IgG4-related Hashimoto’s thyroiditis tend to be young men with shorter duration of the disease compared with those with the non-IgG4-associated subtype of Hashimoto’s disease (51).

### Kidney

IgG4-related kidney disease (IgG4-RKD) usually becomes clinically obvious when there is renal dysfunction or discovered incidentally as multiple low-density lesions on imaging (52). Common findings of IgG4-RKD include tubulointerstitial nephritis (TIN) and membranous glomerulonephritis (MGN). TIN has the typical histologic pattern of IgG4-RD with tubular destruction and atrophy. The presence of IgG4-positive plasma cells is sensitive but not specific for IgG4-TIN. IgG4-bearing plasma cells have been described in necrotizing glomerulonephritis, diabetic nephropathy, lupus nephritis, MGN, and idiopathic TIN (52). Features that can help differentiate IgG4-TIN from other entities include storiform pattern of fibrosis, well-demarcated margins of involvement, and lymphoplasmacytic cell infiltration into and beyond the renal capsule, which is reflected occasionally as a rim-like lesion of the kidney noted on CT (53). MGN is part of the IgG4-RD spectrum and needs to be differentiated from primary MGN. A primary marker of primary MGN, anti-M-type phospholipase A2 receptor antibody, is typically negative in IgG4-MGN (54). In contrast to primary MGN, granular C1q deposits are sometimes prominent, and concurrent IgG4-TIN can occur in IgG4-MGN. Other glomerular diseases involved in IgG4-RKD include IgA nephropathy, IgA vasculitis, and membranoproliferative glomerulonephritis (55).

### Retroperitoneum

IgG4-related retroperitoneal fibrosis (IgG4-RRPF) is characterized by chronic inflammatory and infiltration of fibrotic tissue in the periaortic or periiliac retroperitoneum that encases adjacent structures (56). Different locations of involvement result in varying clinical manifestations. Kidney and urinary tract damage causes hydronephrosis, backache, and acute renal failure. Sclerosing mesenteritis can result from compression on the inferior vena cava, which may cause clinical symptoms of abdominal pain, diarrhea, intestinal obstruction, and ischemia (56). IgG4-related periaortitis and periarteritis are the most common lesions affecting various medium- to large-sized arteries, including the abdominal aorta, iliac artery, renal artery, and mesenteric artery (57). Inflammatory aneurysms are common complications of IgG4-related aortitis, which may present with a pulsatile mass, low-grade fever, back pain, or aneurysm rupture. IgG4-RRPF and idiopathic retroperitoneal fibrosis have similar clinical and imaging features. The two can be distinguished by the presence of classic IgG4-related histopathologic findings and elevated serum IgG4 concentration (58).

### Lymph Nodes

IgG4-related lymphadenopathy (IgG4-RL) may be seen incidentally in excisions made for extranodal disease or detected in patients with symptomatic systemic IgG4-RD (59). IgG4-RL can be generalized or localized and may be the only manifestation of IgG4-RD. The lymph nodes are usually painless and 1–5 cm in size. The frequent sites of involvement include the cervical, supraclavicular, mediastinal, pulmonary-hilar, abdominal, axillary, or inguinal groups. Diagnosis of IgG4-RD in lymph nodes can be difficult because fibrosis is infrequent and typically seen in pseudotumor-like lymph nodes (5). Differential diagnosis of IgG4-RL should include infections, lymphoma, carcinoma, and Castleman’s disease.

### Lungs

Clinical symptoms observed in patients with IgG4-related lung disease are nonspecific and include cough, chest pain, and dyspnea (60). Low-grade fever and weight loss may occur but are often uncommon. Pulmonary parenchymal involvement can manifest as a pseudotumor on imaging and account for some cases previously diagnosed as inflammatory pseudotumor (plasma cell granuloma) in the lungs (61). Interstitial lung disease is another tissue manifestation of IgG4-related lung disease, and the disease may closely resemble nonspecific interstitial pneumonia, organizing pneumonia, or lymphoid interstitial pneumonia (62). Given that these patterns are not specific, histopathologic examination remains critical to diagnosis.

### Gastrointestinal Disease

**Figure 2:**
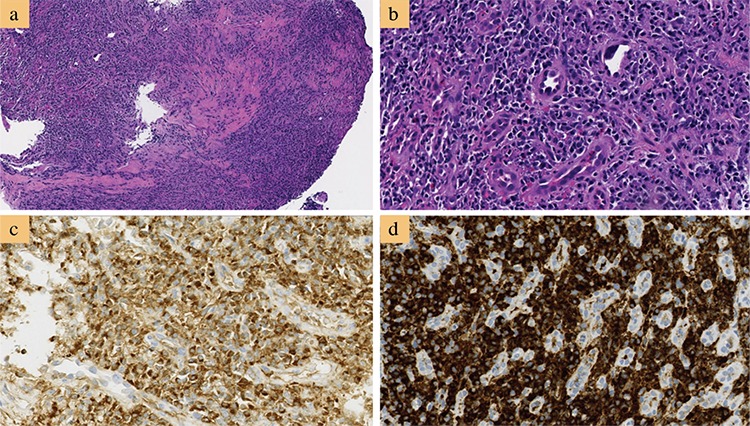
Histologic examination of one classic immunoglobulin G4-related esophagitis. Histologic section shows dense lymphoplasmacytic inflammation rich in plasma cells with storiform fibrosis and obliterative phlebitis (hematoxylin and eosin) stain, a) 40×; b) 400×. Majority of the plasma cells are positive for IgG, c) and immunoglobulin G4, d) (immunohistochemistry, 400×, each.). Reprinted from Obiorah et al. (64). Reprinted with permission of Oxford University Press.

**Figure 3:**
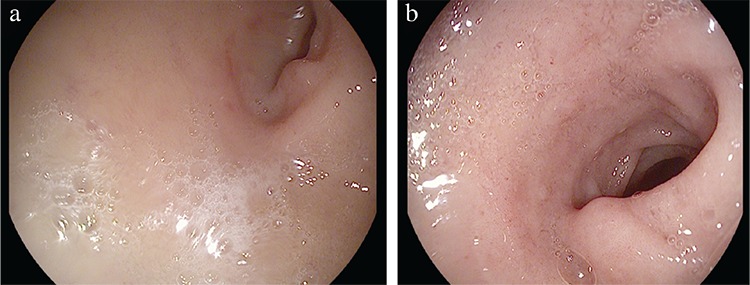
Endoscopy of immunoglobulin G4-related esophageal stricture. a) Stricture of the esophagus before treatment. b) Improvement of the strictured esophagus after 4 months of steroid therapy. Reprinted from Obiorah et al. (64). Reprinted with permission of Oxford University Press.

IgG4-related gastrointestinal disease is a newly evolving disorder in the IgG4-RD family. The clinical manifestations of IgG4-related gastrointestinal disease can either present as a marked thickening of the gastrointestinal wall consisting of marked fibrosis with increased infiltration of IgG4-bearing plasma cells or as an IgG4-related pseudotumor (63). IgG4-related esophagitis is a rare entity. The most frequent symptom is dysphagia, and esophageal stricture is the most common finding via endoscopy (64). Patients with idiopathic chronic esophagitis can be difficult to differentiate from IgG4-related esophagitis because they have similar manifestations such as strictures (64). Thus, histopathologic examination is essential for the diagnosis of IgG4-related esophagitis (Figure 2). Although uncommon, IgG4-related esophagitis should be considered in any case of unexplained stricture in the absence of malignancy. IgG4-related gastritis can show diffusely thickened gastric mucosa on endoscopy or present as submucosal pseudotumors. Esophageal strictures due to IgG4-RD (Figure 3a) have been shown to be responsive to corticosteroid therapy (Figure 3b). Rarely, IgG4-related gastritis can present as refractory gastric ulcers that are unresponsive to *Helicobacter pylori* treatment (65). Polypoid or mass-like lesions are common findings in IgG-RD involving the major duodenal papilla and colon (63). Malignancy should be ruled out, especially in the absence of another organ’s involvement, to avoid unnecessary resection.

### Neurological Involvement

IgG4-RD has been infrequently reported in the central nervous system, and it has a specific propensity for the involvement of the meninges and cranial nerves. IgG4-related hypertrophic pachymeningitis can cause localized or diffuse thickening of the dura mater (66). Frequent symptoms at presentation include headache, cranial nerve palsies, vision disturbances, motor weakness, limb numbness, sensorineural hearing loss, neck stiffness, and seizures. Involvement of cranial nerves usually results from adjacent mass-like lesions (67). Cerebrospinal fluid analysis is often nonspecific and cannot effectively differentiate IgG4-related pachymeningitis from other forms of inflammation. Histologic examination of the meninges is the gold standard for the diagnosis. Clinical manifestation of IGg4-RD involving the pituitary gland depends on the size and location of the lesion within the gland. Therefore, IgG4-related hypophysitis can result in hormone deficiencies from both the anterior and posterior pituitary (68).

### Other Organs

Skin can be involved in IgG4-RD. Two main cutaneous lesions are erythematous plaques and subcutaneous nodules. Other lesions such as brown hyperpigmented papules in patients with dark pigmented skin occur less commonly (69). Typical sites affected include the skin of the head and neck region, and the less affected regions are the trunk and limbs. Involvement of the prostate has also been reported, usually as a presumptive diagnosis based on the presence of IgG4-RD in other organs and resolution of an apparent benign prostatic hypertrophy following glucocorticoid treatment (66). However, biopsy-proven mass-forming IgG4-related prostatitis has also been reported (66). IgG4-related mastitis has been described in five cases and tends to present as painless mass lesions (66,70). Testicular involvement by IgG4-RD can occur as a paratesticular pseudotumor or epididymo-orchitis (71).

## DIAGNOSIS

### Laboratory Parameters

The diagnosis of IgG4-RD depends on the combination of clinical, radiological, pathological, and laboratory modalities including serology. Although quantification of the serum IgG4 concentration is included in all IgG4-RD diagnostic guidelines, approximately one-third of patients with biopsy-proven IgG4-RD have normal serum IgG4 concentrations; thus, serum IgG4 concentration is not required for the diagnosis of IgG4-RD (6). Besides, increased serum IgG4 levels have been observed in patients with a variety of other diseases including primary sclerosing cholangitis (32), making it an insufficient single diagnostic tool. Increased serum IgG4 (typically >135 mg/dL) identifies patients with an active form of the disease, which is correlated with increased concentrations of inflammatory serum biomarkers and hypocomplementemia, increased number of organs affected by the disease, and extensive organ involvement (72). These patients appear to have a shorter time to disease relapse than patients with IgG4-RD with normal serum IgG4 at the time of diagnosis. Serum IgG4 levels usually decrease with glucocorticoid therapy, but they are not disease-specific (73). Some patients with IgG4-RD may remain in remission despite having persistent elevated serum IgG4 levels (6). Elevated circulating plasmablasts have been observed in patients with IgG4-RD (74). The increased levels of plasmablasts correlate with disease activity even in the presence of normal serum IgG4 levels. Increased circulating plasmablasts appear to be superior to serum IgG4, but their use as biomarkers of disease activity is still poorly characterized; further studies are needed before their broad use can be endorsed. In certain cases of IgG4-RD, especially those involving the kidney, complement levels are a useful indicator of disease activity. Hypocomplementemia has been observed at the time of relapse in patients with IgG4-related TIN (53).

### Histology

**Figure 4:**
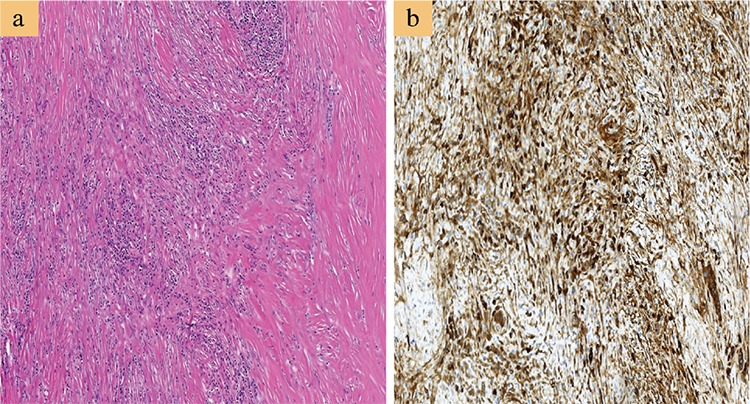
Histologic examination of a case of immunoglobulin G4-related pancreatitis. a) Histologic section shows dense lymphoplasmacytic inflammation rich in plasma cells with storiform fibrosis (hematoxylin and eosin stain, 100×). Majority of the plasma cells are positive for immunoglobulin G4 (D; immunohistochemistry, 200×).

The current diagnostic criteria from the 2012 consensus statement (9) for the histopathologic features of IgG4-RD include a triad of dense lymphoplasmacytic infiltrate, fibrosis arranged at least focally in a storiform manner (Figure 4a), and obliterative phlebitis in the background of increased numbers of IgG4-positive plasma cells (Figure 4b). In most cases, a confident pathological diagnosis of IgG4-RD can be made in the presence of two of the three major histological features. Other histopathologic features, though neither sensitive nor specific, that can be found in IgG4-RD include phlebitis without obliteration of the lumen and elevated numbers of eosinophils. In addition, the presence of >10 IgG4+ plasma cells on biopsy material has been proposed as one element of a comprehensive diagnostic panel. However, the appropriate cutoff point may vary depending on the organ involved and based on the predominance of fibrosis at the time the diagnosis is made. For instance, cases of IgG4-RRPF will have extensive areas of dense fibrosis, which may make identification of IgG4-positive plasma cells challenging (58). Hence, the IgG4/IgG ratio is a powerful tool in establishing a diagnosis of IgG4-RD. Studies (24,66) have suggested an IgG4/IgG plasma cell ratio of >40% as a comprehensive cutoff value in any organ, and this ratio is generally adopted as a histological diagnostic criterion for IgG4-RD. Certain disorders exhibit elevated levels of IgG4-bearing plasma cells in tissue specimens such as primary sclerosing cholangitis, anti-neutrophil cytoplasmic antibody-associated vasculitis, rheumatoid arthritis, inflammatory bowel disease, rhinosinusitis, Rosai-Dorfman disease, autoimmune atrophic gastritis, and multicentric Castleman’s disease (9). However, these conditions all lack the classic characteristic histopathological features of IgG4-RD.

### Clinical Diagnostic Criteria

Although the histopathological findings can provide strong supportive evidence for the diagnosis of IgG4-RD, correlation with the clinical presentation and radiographic findings is often required to make a definitive diagnosis. Some organ-specific diagnostic criteria exist for certain organs such as IgG4-related sclerosing cholangitis (31), but confusion may present when IgG4-RD affects a rare organ for which diagnostic criteria are non-existent. No validated diagnostic criteria exist for IgG4-RD, but the diagnosis should be based on the following characteristics (20,24): (a) mass lesions in one or more organs; (b) lymphoplasmacytic infiltrate, fibrosis, and obliterative phlebitis with >40% of IgG+ plasma cells being IgG4+ and >10 cells/high-power field of biopsy sample; and (c) serum IgG4 concentration >135 mg/dL. A *definite* diagnosis is considered when all three criteria (a-c) are met. The diagnosis of IgG4-RD is considered *probable* when (a) and (b) are met. When (a) and (c) are present, it is deﬁned as *possible* diagnosis. Treatment is generally advocated in patients with either a definite and probable diagnosis especially in patients with an active disease.

## TREATMENT

### Corticosteroid Therapy

Most patients with IgG4-RD respond to corticosteroids, which remain the first line of treatment. A suggested treatment scheme based on the international consensus guidance statement (20,75) on the management of IgG4-RD includes induction therapy with 40 mg of prednisone daily with assessment for treatment response at 2–4 weeks using clinical, radiologic, and biochemical markers. Treatment should be tapered if response is good; otherwise, burnt out disease or an alternate diagnosis should be considered. Tapering of prednisone should be done over 3–6 months to 5 mg daily (6). Maintenance therapy should be considered if the patient has multiorgan disease, significantly elevated serum IgG4, prior history of relapse, and system-specific markers. Maintenance treatment can include low dose of prednisone (2.5–5 mg/day for up to 3 years) or the use of a glucocorticoid sparing agent such as azathioprine (20,75). The response to steroid therapy varies depending on the involved organs and the degree of fibrosis (6). AIP generally responds to glucocorticoids, and pancreatic function can improve following treatment (27,29). By contrast, IgG4-RRPF is less amenable to corticosteroid treatment (56).

### Corticosteroid Sparing Agents

Conventional corticosteroid sparing drugs, such as azathioprine, mycophenolate mofetil, and methotrexate, are widely used either to achieve additional immunosuppression or as steroid sparing agents (76). Both prospective and retrospective studies show that corticosteroids are initially effective for most patients, but they are often poorly tolerated, and disease relapses during or following tapering of corticosteroids are common (75). Although clinical improvement has been described in patients treated with these agents (27,64), none has been tested in prospective, controlled studies. Hart et al. (77) retrospectively compared the results of treatment of patients with relapsing AIP with azathioprine, mycophenolate mofetil, and 6-mecaptopurine with corticosteroid therapy, and they found that the relapse-free survival did not significantly differ between the two groups. Therefore, further evidence for the efficacy of conventional steroid sparing agents in IgG4-RD is needed.

### Rituximab

Rituximab, an anti-CD20 monoclonal antibody, induces B-cell depletion to result in a decrease in IgG4-positive plasma cells and serum IgG4 (77,78). In addition, IgG-positive plasmablasts rapidly decline following treatment with rituximab in patients with IgG4-RD (74). Reports from case series indicate that B-cell depletion with rituximab might be an effective therapy for treating IgG4-RD, but this type of treatment was used in patients who did not respond to corticosteroids, conventional steroid-sparing agents, or both (77,78). Carruthers et al. (79) evaluated the efficacy of rituximab in an open-label pilot trial, in which 83% of 30 patients received rituximab monotherapy. Disease responses were observed in 97% of patients, and the primary outcome achieved by 23 of 30 patients revealed that 47% were in complete remission at 6 months, which was sustained at 12 months in 40% of the patients. Although rituximab shows tremendous promise in the treatment of IgG4-RD, further evaluation is required in the setting of large controlled clinical trials (80).

IgG4-RD is a widely recognized systemic disorder that exhibits pathological features, which remain consistent in any affected organ. Substantial progress has been achieved in the diagnosis and management of IgG4-RD, but many questions remain unanswered in determining the pathogenesis that lies behind the disease, which may be the key to the development of an effective targeted therapy. The clinical manifestations of the disorder are usually nonspecific and vary from organ to organ; thus, detection in early stages can be challenging. Irreversible fibrosis and organ failure can develop if untreated, so clinicians in various specialties should be aware of the disease. The diagnostic approach is complex and requires a combination of clinical, laboratory, and radiological examinations with typical pathological findings. Corticosteroids remain the first line of therapy in active IgG4-RD, but adverse effects of the drug and disease recurrences on reduction or termination of therapy highlight the need for favorable therapy. Rituximab appears to be a promising agent in the treatment of IgG4-RD, but its efficacy needs to be evaluated in large clinical controlled trials.

## Figures and Tables

**Table 1:**
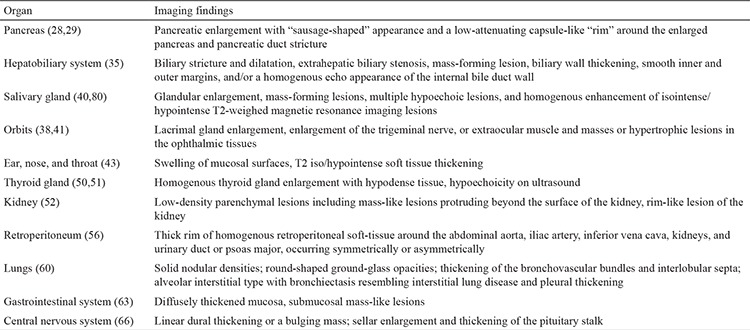
Radiological features of immunoglobulin G4-related disease in major organs
